# Morphologic analysis of the 1st and 2nd tarsometatarsal joint articular surfaces

**DOI:** 10.1038/s41598-023-32500-z

**Published:** 2023-04-20

**Authors:** Melissa R. Requist, Tim Rolvien, Alexej Barg, Amy L. Lenz

**Affiliations:** 1 Department of Orthopaedics, University of Utah Spencer Fox Eccles School of Medicine, Salt Lake City, UT 84108 USA; 2 Department of Trauma and Orthopaedic Surgery, University Medical Center Hamburg-Eppendorf, 20251 Hamburg, Germany; 3 Department of Biomedical Engineering, University of Utah, Salt Lake City, UT 84108 USA; 4 Department of Mechanical Engineering, University of Utah, Salt Lake City, UT 84108 USA

**Keywords:** Bone, Biomedical engineering

## Abstract

Tarsometatarsal joint arthrodesis is used to treat a variety of injuries and deformities in the midfoot. However, the surgical technique has not been optimized, in part due to limited knowledge of morphologic features and variation in the related joints. Previous research has relied primarily on dissection-based anatomical analysis, but quantitative imaging may allow for a more sophisticated description of this complex. Here, we used quantitative micro-CT imaging to examine dimensions, distance maps, and curvature of the four articular surfaces in the first and second tarsometatarsal joints. Image segmentation, articular surface identification, and anatomic coordinate systems were all done with semi or fully automatic methods, and distance and size measurements were all taken utilizing these anatomic planes. Surface curvature was studied using Gaussian curvature and a newly defined measure of curvature similarity on the whole joint and on four subregions of each surface. These data show larger articular surfaces on the cuneiforms, rather than metatarsals, and define the generally tall and narrow articular surfaces seen in these joints. Curvature analysis shows minimally curved opposing convex surfaces. Our results are valuable for furthering knowledge of surgical anatomy in this poorly understood region of the foot.

## Introduction

The surgical anatomy of the first and second tarsometatarsal (TMT) joints is poorly characterized, but is relevant to many operations in the midfoot, most notably correction of both bony and ligamentous injuries to the Lisfranc complex^1–4^. Surgical treatment of these injuries primarily consists of either open reduction and internal fixation (ORIF) or primary arthrodesis^5–8^. Arthrodesis procedures in other joints are known to be influenced by the morphological variation in those joints between individuals^9–11^, but there is little existing research characterizing the typical bony morphology of the joints within the Lisfranc complex, which may partially explain the significant learning curve for this procedure^12^. When treating with ORIF, there is little room for misalignment in this complex as changes in joint contact error can lead to rapidly-developing osteoarthritis^3^. Improved understanding of the bony anatomy of the Lisfranc complex is necessary for refining these surgical methods.

Perhaps the most detailed description of the anatomy of the four bones involved in the first and second tarsometatarsal joints is given in Saraffian’s Anatomy of the Foot and Ankle, which uses dissection and existing literature to discuss the surfaces of each bone and the shapes and curvature of articular surfaces^13^. However, this approach is limited in its ability to quantify curvature, numerically define size and shape, or compare these measurements across opposing joint surfaces. Additionally, new research suggests that dissection-based methods alone are not enough to effectively teach and learn anatomy for successful application of knowledge in a surgical setting^14^. Existing studies in this area have primarily focused on defining mechanisms of injury^15,16^, radiographic injury identification^17–19^, and ligamentous anatomy^20^. Prior research on Lisfranc bony anatomy has examined the average distance between joints using weight-bearing CT in adults with cavovarus alignment^21^ and using plain radiographs in the pediatric population^22^. These showed increased distance in the TMT joints in a cavovarus foot, and a decrease in the distance between the medial cuneiform and second metatarsal with age up to the age of six. Because of its importance to TMT arthrodesis, another study examined the dorsal-plantar height of each of the TMT joints, finding average heights of 32.3 mm and 26.9 mm in the first and second TMT joints, respectively, and a significant correlation between shoe size and TMT joint height^23^.

The goal of this study was to use micro-CT imaging to quantitatively measure the size, curvature, and shape of the articular surfaces that make up the first and second TMT joints and to examine differences in these parameters between opposing joint surfaces. This work can be used to improve surgical techniques in Lisfranc injury treatment and TMT arthrodesis, and to generate hypotheses for future studies in shape modeling in this region.

## Methods

### Imaging and segmentation

Twenty-four samples each of the first and second metatarsal and medial and intermediate cuneiform were imaged by micro-CT, an imaging technology frequently used to examine morphology of small bones in human^24–27^ and animal^28–31^ models. These bones, with the exception of specimen 10, were dissected from 12 bilateral cadaveric specimens (age: 43.8 ± 15.7 years; BMI: 25.1 ± 4.2; all male). Specimen 10 was imaged intact to produce images with the appropriate anatomical positioning. All imaging included a hydroxyapatite imaging phantom and used micro-CT scan parameters of 0.148 mm^3^ resolution, 90 kV tube voltage, and 200 μA tube current (Perkin Elmer Quantum GX2 microCT Imaging System). The calibration phantom was segmented semi-automatically using pre-determined thresholds in Mimics (Materialise, Leuven, Belgium). Regression equations generated from this calibration phantom were used to define thresholds for image segmentation. All four bones were segmented using this semi-automatic method developed in Mimics for this anatomy^32,33^. Once segmented, three-dimensional parts were exported for further analysis based on the automatic algorithm in Mimics. No additional smoothing or decimation was done in order to keep the finest mesh and retain the most image data. This proprietary image to mesh algorithm has been used frequently in foot and ankle biomechanics^34–36^.

### Articular surface identification

**Figure 1:**
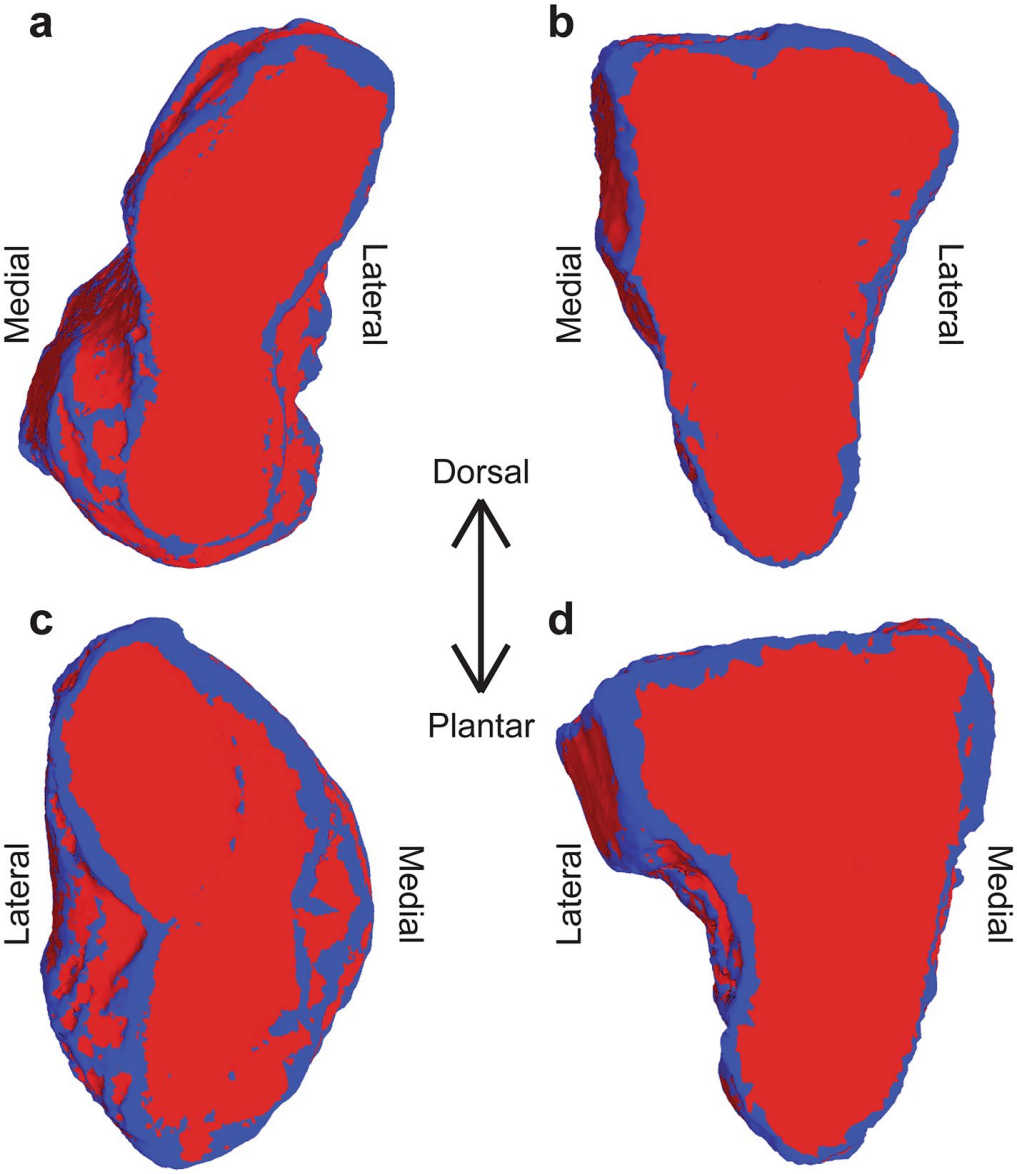
Second-principal curvature identification of the distal articular surfaces of the (**a**) medial cuneiform and (**b**) intermediate cuneiform and proximal articular surfaces of the (**c**) first metatarsal, and (**d**) second metatarsal. Blue shows areas with second-principal curvature below the threshold value and red shows values above that threshold.

Articular surfaces were identified mathematically using second-principal curvature^37^. The curvature was applied to the surface (PostView software v2.10, FEBio Software Suite, University of Utah, Salt Lake City, UT) and consistent thresholds were applied to define each surface as an area of low curvature within a surrounding area of high curvature (Fig. 1). In the medial cuneiform and first metatarsal, a threshold of − 0.25 1/mm was used and in the intermediate cuneiform and second metatarsal, a threshold of − 0.35 1/mm was used. These consistent thresholds allowed for automatic selection of the articular surfaces without requiring manual input, and the values used for each bone followed previously validated methods^32^. Flatter areas showed second-principal curvature greater than the threshold value and more curved areas showed values lower than the threshold. The flat area within a curved boundary was selected, exported, and interior holes were filled with an automatic algorithm to create 3-dimensional parts of the articular surfaces. This method was used to identify the proximal and distal articular surfaces of each of the cuneiforms and the proximal surface of each metatarsal. Because micro-CT gathers data on bone structure and poorly captures cartilage, the articular surfaces in this analysis refer to the subchondral bone at the surface of each bone, not to the articular cartilage.

### Anatomical coordinate systems

Anatomical coordinate systems were applied automatically using the mathematical relationships between articular surfaces, similarly to existing coordinate systems in this anatomy^33^. In the cuneiforms, the proximal–distal axis was defined as the line between the centers of gravity of the proximal and distal joint surfaces, with the center of gravity of the distal surface serving as the origin of the coordinate system since the distal surface was analyzed in this study. Extrema analysis was used to find the most dorsal points of the medial cuneiform surfaces and most plantar points of the intermediate cuneiform surfaces based on the defined proximal–distal axis. The dorsal-plantar axis was defined as the line through the origin parallel to the line from the midpoint between the two centers of gravity to the midpoint between these extrema. The medial–lateral axis was defined as the line perpendicular to both existing axes that intersects the origin. In the metatarsals, the proximal–distal axis was defined as the line from the center of gravity of the proximal articular surface to the center of gravity of the bone object, since only the proximal 5–6 mm of bone fit within the field-of-view of the micro-CT image. The center of gravity of the proximal articular surface served as the origin of the coordinate system. Extrema analysis was used to identify the most lateral points on the plantar and dorsal halves of the first metatarsal proximal surface and most plantar point on the proximal surface of the second metatarsal based on the defined proximal–distal axis. The line between the origin midpoint between the two lateral extrema was used to define the medial–lateral axis in the first metatarsal, and the dorsal-plantar axis was defined as the line through the origin perpendicular to the two existing axes. In the second metatarsal, the line from the origin to the plantar extrema was used to define the dorsal-plantar axis, and the medial–lateral axis was defined as the line through the origin perpendicular to the other two axes. In each case, centers of gravity and extrema analyses in the anatomic coordinate system were used to identify consistent locations for coordinate system application without manual input.

### Articular surface size

**Figure 2:**
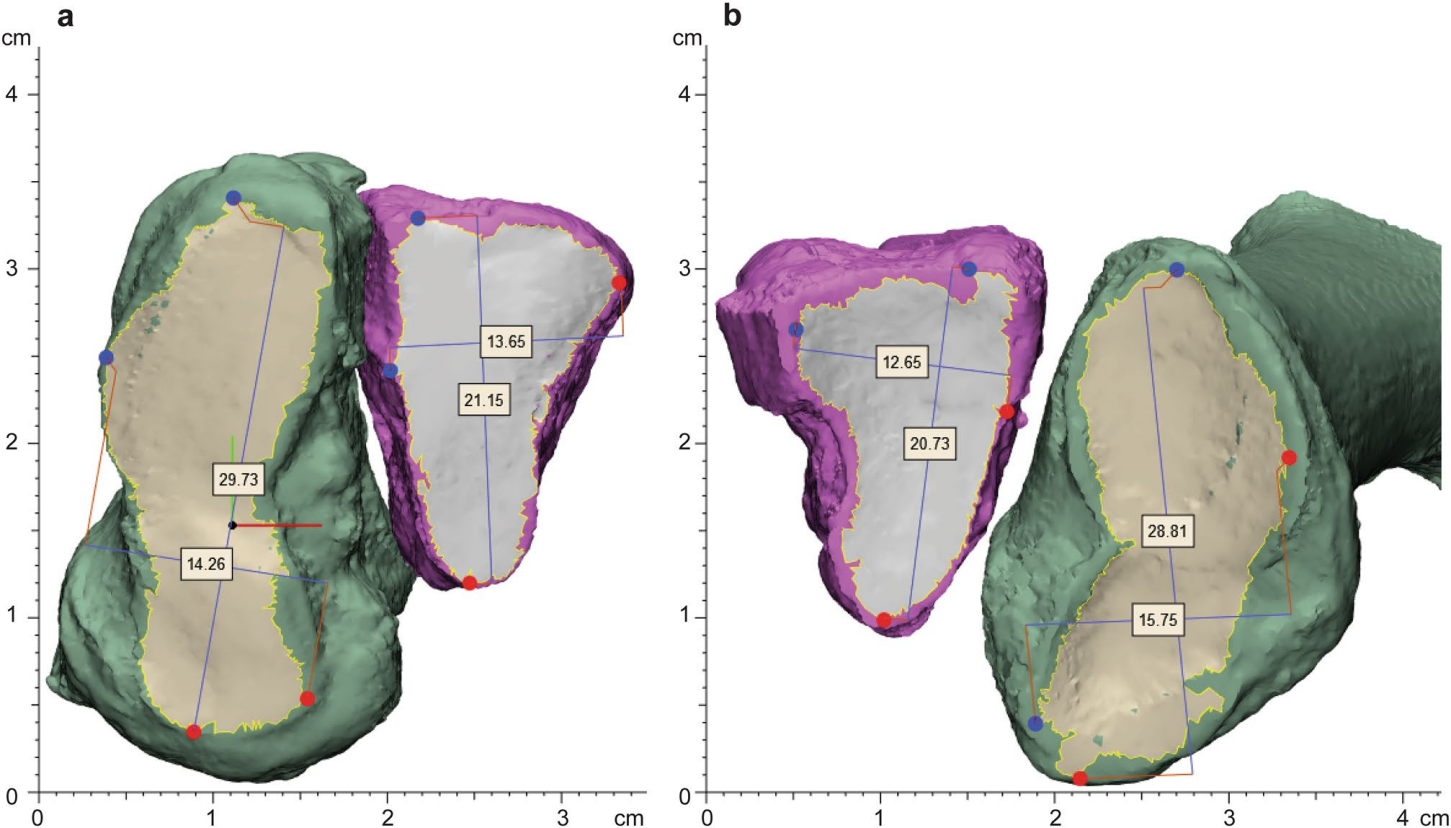
Example of articular surfaces on three-dimensional parts showing the extrema distance measurements (mm), in the anatomic coordinate system, in the (**a**) medial and intermediate cuneiform and (**b**) first and second metatarsal. The medial cuneiform and first metatarsal are shown in green with tan articular surfaces and the intermediate cuneiform, and second metatarsal are shown in pink with grey articular surfaces.

Measurements of articular surface size consisted of surface area, maximum height, and maximum width. Surface area was exported from three-dimensional parts of each articular surface. Maximum height and width were measured by running an extrema analysis in the anatomic coordinate system to identify the furthest dorsal, plantar, medial, and lateral points based on the anatomic axes. Maximum width was defined as the distance parallel to the medial–lateral axis from the furthest medial point to the furthest lateral point. Maximum height was defined as the distance parallel to the proximal-dorsal axis from the furthest proximal point to the furthest dorsal point (Fig. 2).

### Articular surface shape

To create maps of average articular surface shape, width medially and laterally of the dorsal-plantar axis were measured at varying heights every 2 mm plantar and dorsal of the origin of the articular surface. 2 mm was chosen as the distance because it is the approximate thickness of the saw blade used in TMT arthrodesis and was thus the most relevant to understanding the surgical anatomy of this complex. Measurements were made from a projection of the contour of the articular surface onto a plane parallel to the frontal plane intersecting the center of gravity of the articular surface, which was used as the origin. Points were created at the intersection of this contour with lines in the plane parallel to the medial–lateral axis spaced every 2 mm dorsal and plantar of the origin. Medial and lateral distances were calculated from the dorsal-plantar axis to these points. Articular surfaces were different heights, resulting in less than the full 24 measures at the furthest dorsal and plantar heights. Even so, we chose not to normalize heights because the surgical equipment used is the same size regardless of patient size^38–40^ so actual numerical values are more meaningful clinically than normalized heights.

### Articular surface curvature

Average Gaussian curvature for each articular surface was calculated with a fitting radius of 10 mm. This radius was chosen because it is approximately half the height of the articular surface. Gaussian curvature is calculated by multiplication of the principal curvatures of a surface with radius greater than 1. A Gaussian curvature value of zero corresponds to a flat surface. Positive values correspond to a surface that is overall more greatly convex, and negative values correspond to overall concave surfaces^41,42^. Values with magnitude 1 correspond to a perfect sphere. Gaussian curvature is a commonly used measure in morphology analysis, as it is also used to calculate congruence between paired surfaces^37^. The articular surfaces were then cut along the sagittal and transverse planes to yield four subregions of each surface: dorsal-medial, plantar-medial, dorsal-lateral, and plantar-lateral. The average Gaussian curvature for each of these subregions on each surface were also calculated with a fitting radius of 10 mm.

To examine curvature similarity between opposing articular surfaces within each TMT joint, we defined a measure of curvature similarity shown in Eq. (1) where one corresponds to equal magnitudes with matched curvature (one concave and one convex surface) and negative one corresponds to equal magnitudes with mismatched curvature (two concave or two convex surfaces). In a congruent joint, we would expect one concave and one convex surface with similar curvature magnitudes which would be represented by a curvature similarity close to one. A curvature similarity value closer to zero shows a greater difference in magnitude of curvature. Curvature similarity values were calculated for each whole joint and each of the four subregions of the first and second TMT joints.

1 $$Curvature\; similarity = \frac{ - 1}{{\left( {1 + \left| {log\left| {\frac{{k_{cun} }}{{k_{met} }}} \right|} \right|} \right)\left( {\frac{{k_{cun} /k_{met} }}{{k_{cun} /k_{met} }}} \right)}}$$

$${k_{cun}{\text{:}}}\,Gaussian\, curvature\, of\, cuneiform\, of\, opposing\, pair$$

$${k_{met}{\text{:}}}\,Gaussian\,curvature\, of\, metatarsal\, of\, opposing\, pair$$

### Statistical analysis

Mean, standard deviation, and 95% confidence intervals (CI) are reported for each measurement. Shapiro–Wilk test for normality was used to confirm normality in a variety of selected measurements. For articular surface shape, t-tests paired by specimen were used to examine differences in surface area, maximum width, and maximum height on opposing joint surfaces in both TMT joints. To examine articular surface curvature, two-tailed one-sample t-tests were used to compare curvature values to a null hypothesis of zero, which would correspond to a flat surface. Additionally, three-way ANOVA was conducted to evaluate differences in curvature between regions, specimens, and side. Games-Howell post-hoc analysis, which corrects for unequal variances^43^, was used to identify significance in region-to-region comparisons. Two-tailed one-sample t-tests were used to compare curvature similarity values to a null hypothesis of one, corresponding to perfectly matched curvature, and negative one, corresponding to perfectly matched curvature. One-tailed one-sample t-tests were used to identify which joints and subregions had curvature similarity significantly greater or less than zero, with direction chosen by the mean value. Three-way ANOVA with Games-Howell post-hoc analysis was used to identify differences in curvature similarity between regions, specimen, and side. All statistical tests used a significance level α = 0.05.

## Results

### Articular surface size

The average surface area measures of the medial and intermediate cuneiform distal articular surfaces were 346.31 mm^2^ and 180.15 mm^2^, respectively, and the first and second metatarsal proximal articular surface area measures were 347.42 mm^2^ and 168.03 mm^2^, respectively. Summary surface area data is presented in Table 1 and raw data is given in supplemental Table A-1. The average ratio of the medial cuneiform distal surface to first metatarsal proximal surface was 1.00 (95% CI 0.97–1.03) and the ratio of the intermediate cuneiform distal surface to second metatarsal proximal surface averaged 1.07 (95% CI 1.03–1.11). Paired t-tests showed no significant difference in surface areas between the medial cuneiform and first metatarsal, but the intermediate cuneiform had significantly greater surface area than the second metatarsal (p = 0.002). All p-values for articular surface size comparisons are given in supplemental Table B-1.

**Table 1:** Surface area and extrema distance measurements for the articular surfaces of each of the four bones in the first and second TMT joints giving mean, standard deviation, and 95% confidence intervals. Width refers to medial–lateral maximum distance and height refers to dorsal-plantar maximum distance.

Bone	Surface area (mm^2^)			Width (mm)			Height (mm)		
	Mean	SD	95% CI	Mean	SD	95% CI	Mean	SD	95% CI
Medial cuneiform	346.31	24.260	325.300–367.317	18.20	1.98	17.41–18.99	29.21	1.63	28.56–29.86
First metatarsal	347.42	47.58	328.690–366.759	17.59	1.58	16.96–18.22	28.53	1.57	27.90–29.16
Intermediate cuneiform	180.15	36.90	165.385–194.912	13.26	1.23	12.77–13.75	21.85	2.03	21.04–22.66
Second metatarsal	168.03	31.44	155.451–180.608	12.48	1.08	12.05–12.91	20.59	2.09	19.75–21.43

Summary width and height data for all articular surfaces are given in Table 1, and raw data is presented in supplemental Table A-2. The medial cuneiform averaged 18.20 mm wide and 29.21 mm tall, and the first metatarsal averaged 17.59 mm wide and 28.53 mm tall. The intermediate cuneiform averaged 13.26 mm wide and 21.85 mm tall, and the second metatarsal averaged 12.48 mm wide and 20.59 mm tall. Paired t-tests showed that each cuneiform surface had significantly greater maximum width (p = 0.022) and height (p = 0.007) measurements than their opposing metatarsal surfaces, respectively. These p-values are given in supplemental Table B-1.

### Articular surface shape

**Figure 3:**
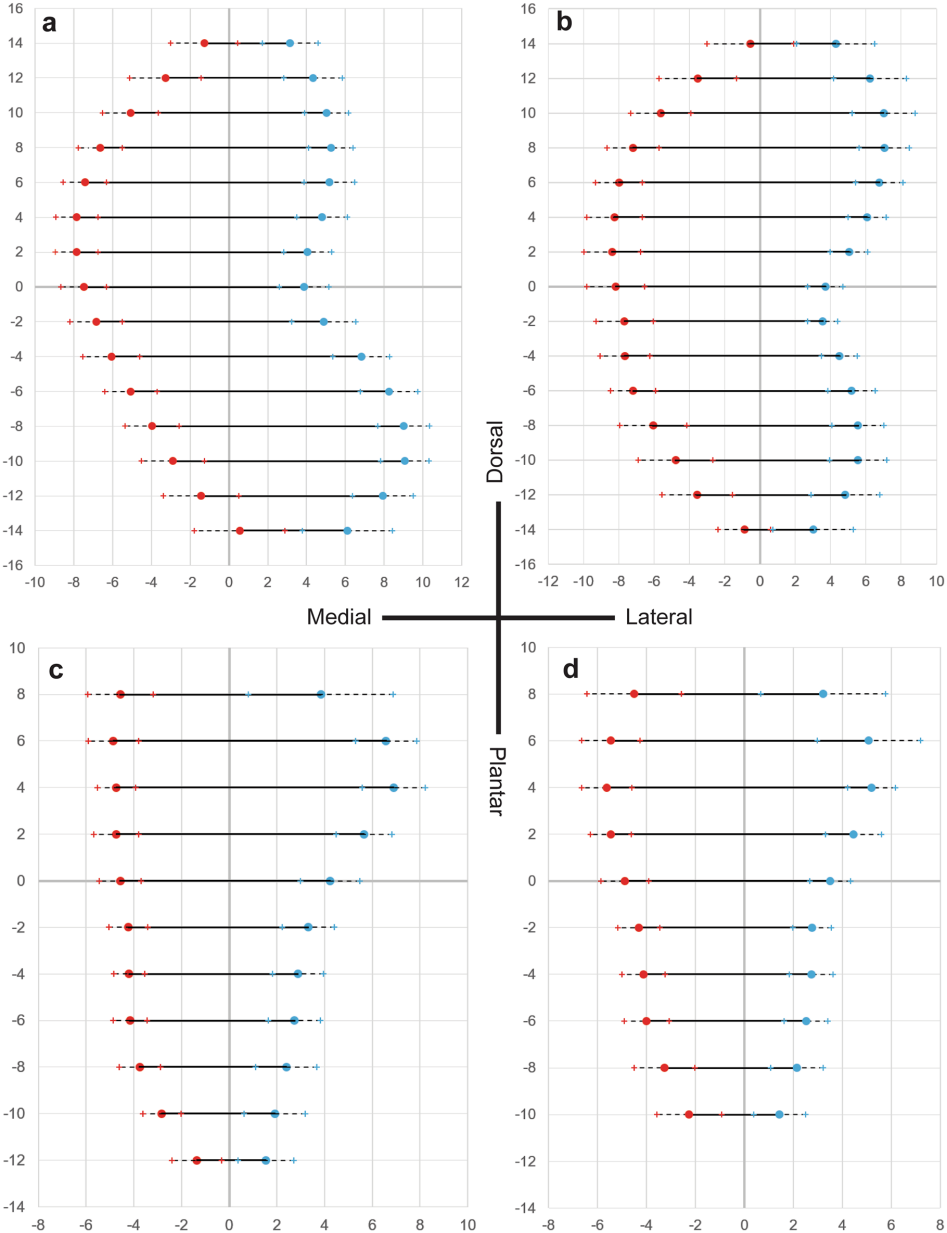
Distance measurements of articular surface depth at varying heights with 2 mm separation in the (**a**) medial cuneiform, (**b**) first metatarsal, (**c**) intermediate cuneiform, and (**d**) second metatarsal. Medial distances (mm) are given in red and lateral distances (mm) given in blue and whiskers show plus and minus one standard deviation for each measure. Only heights with at least 12 measurements are included.

Summary distance measurements at each height in each articular surface are given in Table 2. Average bone shapes, showing mean distance values plus and minus one standard deviation, are shown for each bone in Fig. 3. While all data is included in the table, only heights with a minimum n = 12 were included in the figures. Height measurements with less than half the specimens were excluded from these figures because the reduced sample size skewed the importance of data from certain subjects and overrepresented distance at those heights as the majority of samples did not reach that far dorsal or plantar. Shapiro–Wilk test was used to confirm normality in heights with at least 12 measures. In the medial cuneiform, the total distance at the center was 11.36 mm (95% CI 11.19–11.54 mm). The distance at a height 14 mm plantar of center (− 14 mm), which was the furthest plantar height with at least 12 measurements, was 5.56 mm (95% CI 4.98–6.13 mm). At a height of 14 mm, which was the furthest dorsal height with at least 12 measurements, the average total distance was 4.43 mm (95% CI 4.21–4.65 mm). The first metatarsal had an average total distance at the center of 11.87 mm (95% CI 11.70–12.05 mm). The distance at a height of − 14 mm was 3.90 mm (95% CI 3.59–4.21 mm) and the distance at a height of 14 mm was 4.84 mm (95% CI 4.51–5.18 mm). In the intermediate cuneiform, the average distance at the center was 8.79 mm (95% CI 8.67–8.92 mm). The distance at a height of − 12 mm was 2.89 mm (95% CI 2.66–3.13 mm) and the distance at a height of 8 mm was 8.40 mm (95% CI 8.06–8.75 mm). The second metatarsal had an average total distance at the center of 8.39 mm (95% CI 8.28–8.49 mm). The distance at a height of − 10 mm was 3.69 mm (95% CI 3.52–3.87 mm) and the distance at a height of 8 mm was 7.73 mm (95% CI 7.35–8.11 mm). Raw data for each of these distance measurements are given in supplemental Tables A-3 through A-6.

**Table 2:** Mean, standard deviation, and 95% confidence interval for width measurements at varying heights every 2 mm in each articular surface in the first and second TMT joints. Positive heights are dorsal to the center of the surface and negative heights are plantar.

Height	Medial distance			Lateral distance			Total distance			n
	Mean	SD	95% CI	Mean	SD	95% CI	Mean	SD	95% CI	
Medial cuneiform										
16	2.09	1.95	− 0.11–4.30	1.98	0.32	1.62–2.34	4.07	2.26	2.59–5.55	3
14	1.28	1.73	0.48–2.07	3.15	1.44	2.49–3.82	4.43	2.03	4.21–4.65	18
12	3.29	1.84	2.55–4.02	4.35	1.50	3.74–4.95	7.63	2.49	7.43–7.83	24
10	5.09	1.44	4.51–5.66	5.03	1.14	4.58–5.49	10.12	1.77	9.97–10.26	24
8	6.65	1.13	6.20–7.10	5.26	1.16	4.79–5.72	11.91	1.28	11.80–12.01	24
6	7.44	1.11	6.99–7.88	5.18	1.31	4.66–5.71	12.62	1.70	12.48–12.76	24
4	7.85	1.08	7.42–8.28	4.81	1.31	4.29–5.34	12.66	1.85	12.51–12.81	24
2	7.85	1.10	7.41–8.29	4.06	1.23	3.56–4.55	11.91	2.00	11.74–12.07	24
0	7.49	1.18	7.02–7.96	3.87	1.27	3.37–4.38	11.36	2.12	11.19–11.54	24
− 2	6.85	1.35	6.31–7.39	4.90	1.65	4.24–5.56	11.75	2.70	11.53–11.97	24
− 4	6.06	1.47	5.47–6.65	6.82	1.47	6.23–7.41	12.89	2.60	12.67–13.10	24
− 6	5.06	1.36	4.52–5.61	8.26	1.47	7.67–8.85	13.32	2.48	13.12–13.52	24
− 8	3.97	1.40	3.41–4.53	9.02	1.34	8.48–9.55	12.99	2.18	12.81–13.17	24
− 10	2.89	1.63	2.24–3.54	9.07	1.25	8.56–9.57	11.95	2.13	11.78–12.13	24
− 12	1.44	1.93	0.63–2.25	7.94	1.57	7.28–8.60	9.38	2.75	9.14–9.63	22
− 14	− 0.55	2.34	− 1.82–0.72	6.11	2.32	4.84–7.37	5.56	3.83	4.98–6.13	13
− 16	1.09	–	–	4.94	–	–	6.03	–	–	1
First metatarsal										
16	− 1.88	–	–	2.43	–	–	0.55	–	–	1
14	0.55	2.44	− 0.55–1.65	4.29	2.22	3.30–5.29	4.84	3.23	4.51–5.18	19
12	3.54	2.20	2.66–4.42	6.23	2.05	5.41–7.05	9.77	3.23	9.50–10.03	24
10	5.62	1.70	4.94–6.30	7.01	1.79	6.30–7.73	12.64	2.56	12.43–12.85	24
8	7.19	1.47	6.60–7.78	7.04	1.43	6.47–7.61	14.23	1.97	14.07–14.39	24
6	8.00	1.33	7.47–8.53	6.76	1.34	6.22–7.30	14.76	1.94	14.60–14.92	24
4	8.25	1.56	7.63–8.88	6.07	1.09	5.63–6.50	14.32	1.91	14.16–14.48	24
2	8.38	1.60	7.74–9.02	5.04	1.06	4.62–5.47	13.43	2.20	13.25–13.60	24
0	8.18	1.64	7.52–8.83	3.70	1.01	3.30–4.10	11.87	2.15	11.70–12.05	24
− 2	7.69	1.62	7.04–8.34	3.55	0.86	3.20–3.89	11.23	1.75	11.09–11.38	24
− 4	7.67	1.40	7.11–8.23	4.49	1.02	4.08–4.89	12.15	1.80	12.01–12.30	24
− 6	7.20	1.28	6.69–7.71	5.17	1.35	4.63–5.71	12.37	1.71	12.23–12.51	24
− 8	6.06	1.89	5.30–6.82	5.55	1.47	4.96–6.13	11.61	2.08	11.44–11.78	24
− 10	4.78	2.11	3.94–5.63	5.55	1.62	4.90–6.20	10.33	1.94	10.18–10.49	24
− 12	3.57	2.00	2.71–4.43	4.83	1.94	4.00–5.66	8.40	1.67	8.24–8.56	21
− 14	0.89	1.48	0.05–1.73	3.01	2.28	1.72–4.29	3.90	1.90	3.59–4.21	12
− 16	− 2.29	–	–	3.51	–	–	1.22	–	–	1
Intermediate cuneiform										
10	2.28	2.15	0.79–3.77	1.34	1.16	0.54–2.15	3.62	2.62	2.98–4.26	8
8	4.56	1.37	3.97–5.15	3.84	3.03	2.55–5.14	8.40	3.68	8.06–8.75	21
6	4.85	1.06	4.42–5.29	6.57	1.28	6.05–7.09	11.43	1.93	11.26–11.59	23
4	4.72	0.80	4.41–5.04	6.89	1.32	6.36–7.41	11.61	1.75	11.47–11.75	24
2	4.73	0.94	4.35–5.11	5.65	1.17	5.18–6.11	10.38	1.68	10.24–10.51	24
0	4.57	0.88	4.22–4.92	4.23	1.24	3.73–4.72	8.79	1.54	8.67–8.92	24
− 2	4.23	0.82	3.90–4.55	3.31	1.09	2.87–3.75	7.54	1.41	7.42–7.65	24
− 4	4.20	0.64	3.94–4.45	2.88	1.06	2.46–3.31	7.08	1.49	6.96–7.20	24
− 6	4.15	0.72	3.87–4.44	2.74	1.09	2.30–3.18	6.89	1.41	6.78–7.01	24
− 8	3.75	0.86	3.41–4.10	2.39	1.27	1.88–2.90	6.14	1.71	6.00–6.28	24
− 10	2.82	0.80	2.49–3.15	1.91	1.28	1.39–2.44	4.74	1.57	4.60–4.87	23
− 12	1.36	1.05	0.83–1.89	1.54	1.17	0.94–2.13	2.89	1.78	2.66–3.13	15
− 14	0.87	1.02	− 0.28–2.02	1.14	1.36	− 0.40–2.68	2.01	1.13	1.27–2.75	3
Second metatarsal										
10	3.07	2.61	0.79–5.36	1.19	2.66	− 1.14–3.52	4.26	3.25	2.99–5.54	5
8	4.51	1.94	3.66–5.36	3.21	2.56	2.09–4.33	7.73	3.88	7.35–8.11	20
6	5.45	1.19	4.98–5.93	5.08	2.11	4.24–5.93	10.54	2.72	10.32–10.76	24
4	5.63	1.03	5.22–6.04	5.20	0.98	4.81–5.59	10.83	1.39	10.72–10.94	24
2	5.46	0.84	5.12–5.79	4.46	1.15	4.00–4.92	9.91	1.35	9.80–10.02	24
0	4.89	0.98	4.49–5.28	3.50	0.83	3.16–3.83	8.39	1.27	8.28–8.49	24
− 2	4.31	0.87	3.97–4.66	2.77	0.79	2.45–3.08	7.08	1.25	6.98–7.18	24
− 4	4.12	0.88	3.77–4.48	2.73	0.90	2.37–3.09	6.86	1.17	6.76–6.95	24
− 6	4.00	0.92	3.63–4.36	2.51	0.89	2.16–2.87	6.51	1.31	6.40–6.62	24
− 8	3.26	1.24	2.77–3.76	2.14	1.08	1.71–2.57	5.40	1.65	5.27–5.54	24
− 10	2.26	1.32	1.69–2.82	1.44	1.07	0.98–1.89	3.69	1.84	3.52–3.87	21
− 12	1.23	0.70	0.78–1.69	0.81	0.97	0.17–1.45	2.04	1.22	1.78–2.31	9

### Articular surface curvature

Gaussian curvature measurements for the whole surface showed slightly positive curvature values for all four articular surfaces. This summary data is given in Table 3, and raw curvature data for the whole surface is presented in supplemental Table A-7. One-sample t-tests showed curvature significantly greater than zero in the first and second metatarsals (p = 0.044), but no significant difference from zero in the curvature of the cuneiforms. All p-values are given in supplemental Table B-2. On the whole surface level, the cuneiforms showed a relatively flat curvature, and the metatarsal articular surfaces showed a significantly convex curvature. Three-way ANOVA showed significant differences in curvature between bones (p = 0.033) and specimen (p < 0.001), but not between sides. Games-Howell post-hoc analysis revealed that the second metatarsal surface had a significantly more convex curvature than the surfaces of the medial cuneiform or first metatarsal (p = 0.003), but there were no other significant comparisons. All p-values from this analysis are given in supplemental Table B-3.

**Table 3:** Gaussian curvature measures of the whole surface and each quadrant of the articular surfaces within the first and second TMT joints. Zero represents a flat surface, positive values show convex curvature and negative values show concave curvature.

	Medial cuneiform			First metatarsal		
	Mean	SD	95% CI	Mean	SD	95% CI
Whole bone	0.001	0.016	− 0.006–0.007	0.005	0.024	0.016–0.032
Dorsal-medial	0.008	0.025	− 0.002–0.018	0.008	0.035	− 0.006–0.022
Plantar-medial	0.001	0.035	− 0.013–0.014	− 0.004	0.026	− 0.015–0.006
Plantar-lateral	0.006	0.033	− 0.007–0.020	0.023	0.038	0.007–0.038
Dorsal-lateral	0.021	0.018	0.014–0.028	0.038	0.018	0.030–0.045

	Intermediate cuneiform			Second metatarsal		
	Mean	SD	95% CI	Mean	SD	95% CI
Whole bone	0.010	0.023	0.001–0.019	0.024	0.019	0.016–0.032
Dorsal-medial	− 0.012	0.029	− 0.023–0.000	− 0.030	0.025	− 0.040–0.020
Plantar-medial	−0.005	0.032	-0.017–0.008	− 0.013	0.032	− 0.026–0.000
Plantar-lateral	0.001	0.043	− 0.016–0.008	− 0.008	0.060	− 0.032–0.016
Dorsal-lateral	− 0.011	0.031	− 0.024–0.001	− 0.015	0.104	− 0.056–0.027

All subregions in the medial cuneiform averaged slightly positive Gaussian curvature, but three of the four regions of the intermediate cuneiforms averaged negative curvature values. However, only the dorsal-lateral region of the medial cuneiform surface had a curvature significantly different from zero (p < 0.001), and all other regions of the cuneiform surfaces had curvatures not significantly different from zero, representing a flat surface. Summary curvature data is presented in Table 3 and all raw curvature data is given in supplemental Table A-8. All p-values are given in supplemental Table B-2. Three of the four subregions of the first metatarsal averaged positive Gaussian curvature, but all subregions of the second metatarsal averaged slightly negative curvature measure. The dorsal-lateral and plantar-lateral regions of the first metatarsal had significantly convex curvature (p = 0.009) and the dorsal-medial region of the second metatarsal had significantly concave curvature (p < 0.001). All other regions of the metatarsals were not significantly curved. Three-way ANOVA in the medial cuneiform showed significant differences in curvature between region (p = 0.033) and specimen (p < 0.001), but not between sides. Games-Howell post-hoc analysis revealed that the dorsal-medial region had significantly more convex curvature than either the plantar-medial or plantar-lateral region (p = 0.035). However, three-way ANOVA with post-hoc analysis revealed no significant differences in curvature between subregions in any of the other articular surfaces. All p-values are given in supplemental Table B-4.

In the whole joint and in each of the four subregions in each TMT joint, the average curvature similarity values were negative. This summary data is presented in Table 4, and all raw data is given in supplemental Tables A-9 and A-10. Both the first and second TMT joints, as well as all four regions of each joint, showed curvature similarity values significantly different from 1 (p < 0.001) and from negative 1 (p = 0.001), showing a significant difference from matched or anti-matched surfaces. All p-values are given in supplemental Table B-5. Both whole joints showed curvature similarity significantly less than zero (p = 0.003). Each region of the first TMT joint except the plantar-medial showed curvature similarity significantly less than zero (p = 0.001), and the two plantar regions of the second TMT joint shoed curvature similarity significantly less than zero (p = 0.045). Three-way ANOVA showed no significant difference in curvature similarity between the first and second TMT joints or between subregions within either joint. Games-Howell post-hoc analysis showed that the plantar-lateral region of the second TMT joint had significantly more negative curvature similarity than the plantar-medial region (p = 0.035), but there were no other significant comparisons. All p-values for these comparisons are given in supplemental Table B-6.

**Table 4:** Curvature similarity values for the whole joint and four quadrant subregions of the first and second TMT joints. Positive values show matched curvature, with one concave and one convex surface. Negative values show mismatched curvature, with either two concave or two convex surfaces. Magnitudes greater to one show more similar curvature magnitudes between opposing surfaces.

	1st TMT joint			2nd TMT joint		
	Mean	SD	95% CI	Mean	SD	95% CI
Whole joint	− 0.536	0.556	− 0.76 to − 0.31	− 0.431	0.691	− 0.71 to − 0.15
Dorsal-medial	− 0.443	0.623	− 0.69 to − 0.19	− 0.202	0.790	− 0.52 to 0.11
Plantar-medial	− 0.218	0.717	− 0.51 to − 0.07	− 0.271	0.749	− 0.57 to 0.03
Plantar-lateral	− 0.583	0.617	− 0.78 to − 0.29	− 0.548	0.597	− 0.79 to − 0.31
Dorsal-lateral	− 0.655	0.510	− 0.86 to − 0.45	− 0.255	0.789	− 0.57 to 0.06

## Discussion

Knowledge of the exact dimensions of the TMT articular surfaces is important for complete preparation of the joint surfaces during arthrodesis^44^. However, the exact dimensions of the TMT articular surfaces were not previously available. We here demonstrated that the articular surfaces of the cuneiforms were generally larger than their opposing metatarsal surfaces in width, height, and surface area in the second TMT joint. This information can be used clinically, as appropriate understanding of the dimensions may allow complete surgical preparation of the articular surfaces.

Average heights in all four bones are shorter than previously reported heights for the first and second TMT joints^23^, which is expected because our study focused solely on articular surfaces without considering the height of the surrounding bone. Both cuneiforms showed relatively flat overall curvature, averaging slightly convex curvature across both whole surfaces and slightly concave curvature in most regions of the intermediate cuneiform, which supports previously examined curvature of these surfaces^13^. Proximal articular surfaces on the first and second metatarsals both showed convex overall curvature, although each region of the second metatarsal averaged a slightly concave curvature. The only existing comprehensive description of the morphology of these bones claimed these surfaces to be slightly concave, rather than convex^45^. However, these descriptions were derived from dissection-based methods that lacked measurement analyses, and did not report number of specimens. The paucity of comparable bodies of literature further supports the need for additional studies to characterize midfoot morphology. Nonetheless, the variability between our findings and this existing work indicates that further research is necessary to definitively characterize the curvature of these metatarsal articulations in a larger population.

Curvature similarity in each full TMT joint was lower than zero, showing mismatched curvature. While curvature similarity in each subregion averaged below zero, a few of these regions had 95% confidence intervals including zero, which may indicate that the similarity measure has limited use in relatively flat regions, since a small change in curvature will change the direction of the similarity measure if both curvatures are close to zero. Since each of these full surfaces averaged convex curvature, we can predict that there is a slightly smaller joint space at the center of the joint than on the sides. While curvature was relatively dissimilar, the degree of dissimilarity did not vary in different regions (i.e., quadrants) of the joint. Our description of articular surface shape and width at varying height may additionally assist in surgical planning for TMT arthrodesis. For instance, our findings on comparable curvature dissimilarity between the quadrants could facilitate planning of cartilage resection and/or correction osteotomy in the TMT joint and cartilage removal in TMT arthrodesis with accurate restoration of joint morphology (e.g., length, angle).

**Figure 4:**
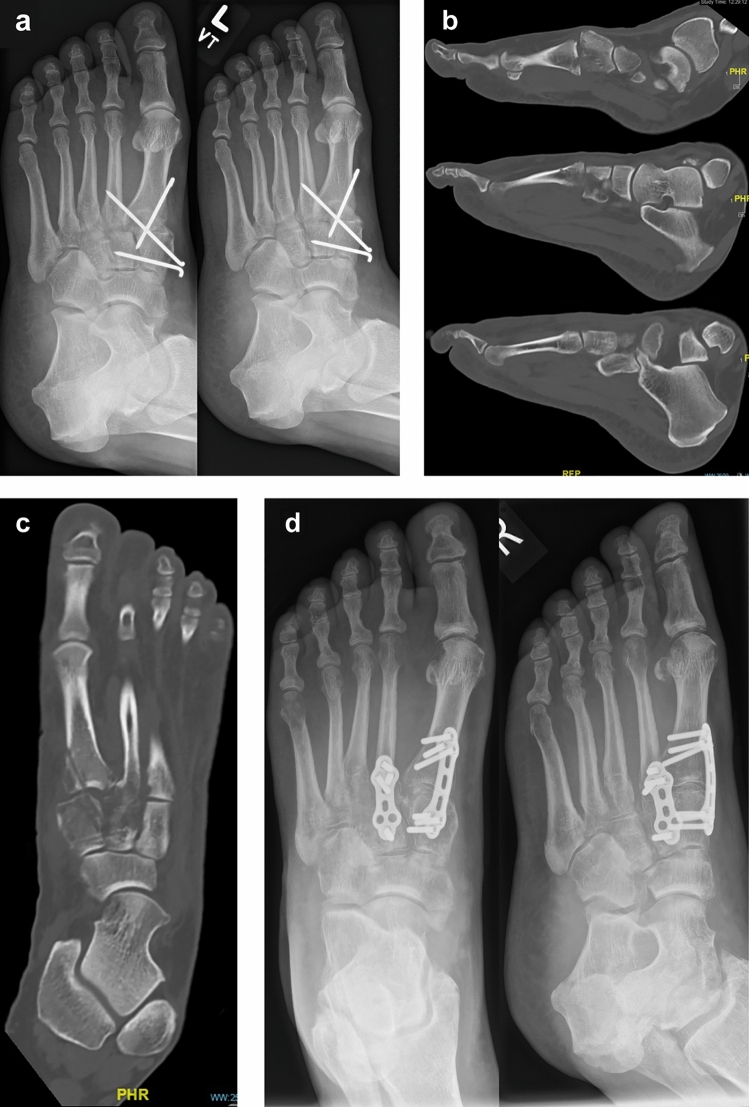
A 39-years-old female patient had a distortion trauma of her right midfoot resulting in a Lisfranc injury involving first and second tarsometatarsal joints. (**a**) She underwent a closed reduction of the Lisfranc joint and transfixation using Kirschner-wires (in an outside hospital). (**b**) Eight weeks later Kirschner wires were removed, and CT scan was performed demonstrating substantial dislocation of the first (top) and second (middle) tarsometatarsal joints. The alignment of the third tarsometatarsal joint was OK (bottom). (**c**) Severe dislocation of the first and second tarsometatarsal joints has been also confirmed using axial CT scan. (**d**) Revision surgery was performed including open anatomic reduction of both dislocated joints and transfixation using two locking plates.

While there have been many surgical techniques described for the Lapidus procedure, all include the common step of cartilage removal on articulating surfaces^38^. This can be done by debriding the surface using curette or removal of bone slice using an oscillating saw. This study showed a relatively low width in the proximal first metatarsal and distal medial cuneiform. Because of this joint line configuration, aggressive sawing without exact knowledge of the anatomy may result in the iatrogenic injury of the shaft of the second metatarsal. This knowledge of joint anatomy may also help decrease complications associated with misaligned fixation (Fig. 4). Beyond the impact of our characterization of surgical anatomy, these data and methods allow for future work in shape modeling, congruence analysis, and patient-specific modeling. Having a quantitative description of this anatomy allows for generation of hypotheses for shape modeling and congruence analysis. Having a basic morphologic characterization may allow for machine-learning based image segmentation and articular surface identification to use in patient-specific surgical planning from clinical CT or weight-bearing CT images^21,46,47^.

Limitations of this study include the use of dissected cadaveric specimens, which prevents any analysis of joint space. Further, all cadaveric specimens used in this study were male, so this may not appropriately define the typical TMT joint anatomy in a female population, especially since females tend to have smaller feet than males and there is an established correlation between TMT joint height and foot size^29^. Some of the variation in distance measurements may be related to centering the origin of the anatomical axis at the center of gravity of the articular surface, and a more sophisticated method to align articular surfaces could provide a clearer picture of the dorsal and plantar ends of the joint surfaces. Another limitation from the use of micro-CT imaging is the lack of information on articular cartilage. While this analysis focuses on the morphology of bony surfaces, additional research using alternative imaging techniques, such as MRI, is necessary to fully characterize the cartilage morphology of these joints. In order to make this study most relevant to surgical procedures, articular surface measurements were not normalized to either bone size or foot size. This allows for better use of this data in surgical planning, but may limit the detail of the explained articular surface morphology by not accounting for overall size as a variable. However, it lays the groundwork for future studies using shape modeling and congruence analysis to further explain the morphology of these joints.

In summary, our data provide the first quantitative data on width, height, surface area, and curvature, which are essential for optimal surgical management of traumatic and degenerative conditions of the Lisfranc complex. These data define the relatively tall and narrow articular surfaces seen in these joints and suggest larger surfaces on the cuneiform surfaces than metatarsals, which may guide joint preparation for TMT arthrodesis. Curvature measures show relatively flat surfaces with generally opposing convex surfaces, which can be explained in greater detail through future work in congruence and shape modeling in this region of the midfoot.

## Supplementary Information

Supplementary Information.

## Data Availability

The data generated during this analysis is available at Zenodo and can be used under the Creative Commons Attribution 4.0 International license. 10.5281/zenodo.7555973.
