# Supplementary material for: Morphologic analysis of the 1st and 2nd tarsometatarsal joint articular surfaces
Source: Sci Rep. 2023 Apr 20;13:6473. doi: 10.1038/s41598-023-32500-z (PMC10119313; doi:10.1038/s41598-023-32500-z)
Supplement: Supplementary file 1 — Supplementary Information. [file 41598_2023_32500_MOESM1_ESM.docx]

**Supplemental Material**

Supplement A: Raw data measurements for each specimen

Table A-1: Surface area measures for each specimen in mm^2^

|  | Medial Cuneiform | First Metatarsal | Intermediate Cuneiform | Second Metatarsal |
| --- | --- | --- | --- | --- |
| 1L | 374.4501 | 346.3424 | 221.8287 | 164.623 |
| 1R | 351.9355 | 343.6554 | 195.7183 | 179.5083 |
| 2L | 365.3996 | 365.7811 | 204.9518 | 199.7794 |
| 2R | 338.906 | 344.53 | 210.5734 | 187.2958 |
| 3L | 351.2922 | 318.0114 | 222.088 | 192.4842 |
| 3R | 304.7144 | 319.3317 | 228.8167 | 200.958 |
| 4L | 372.1666 | 354.5687 | 175.484 | 167.6568 |
| 4R | 324.5302 | 346.1431 | 208.1601 | 171.1166 |
| 5L | 303.9132 | 284.5897 | 143.9177 | 140.8803 |
| 5R | 282.1796 | 314.2178 | 105.2467 | 110.577 |
| 6L | 259.2553 | 339.2443 | 150.9354 | 139.3311 |
| 6R | 324.138 | 312.2477 | 152.204 | 160.3039 |
| 7L | 316.3351 | 309.465 | 148.4534 | 131.3375 |
| 7R | 317.8735 | 343.1103 | 132.656 | 135.2031 |
| 8L | 370.2285 | 362.7762 | 179.5181 | 186.3986 |
| 8R | 372.4457 | 361.6025 | 172.6632 | 182.9243 |
| 9L | 401.7805 | 383.0909 | 231.9219 | 231.2481 |
| 9R | 414.6689 | 379.3663 | 245.9129 | 221.0632 |
| 10L | 297.2101 | 308.4715 | 184.4898 | 155.8017 |
| 10R | 264.5891 | 270.6707 | 175.0729 | 166.4686 |
| 11L | 366.384 | 350.4577 | 145.9244 | 126.9993 |
| 11R | 323.4306 | 340.0205 | 131.3563 | 118.2966 |
| 12L | 465.7567 | 476.3071 | 164.1135 | 172.5812 |
| 12R | 447.8211 | 471.3871 | 191.5578 | 189.871 |

Table A-2: Maximum width and height measures for each specimen in mm

|  | Medial Cuneiform | | First Metatarsal | | Intermediate Cuneiform | | Second Metatarsal | |
| --- | --- | --- | --- | --- | --- | --- | --- | --- |
|  | Width | Height | Width | Height | Width | Height | Width | Height |
| 1L | 19.73 | 29.80 | 18.60 | 29.18 | 14.70 | 22.12 | 12.65 | 18.39 |
| 1R | 17.83 | 30.24 | 17.64 | 27.83 | 13.58 | 21.55 | 13.29 | 23.69 |
| 2L | 20.35 | 28.91 | 19.33 | 28.32 | 15.36 | 22.65 | 14.12 | 22.38 |
| 2R | 18.44 | 28.30 | 17.48 | 27.58 | 14.34 | 22.43 | 13.66 | 20.99 |
| 3L | 16.69 | 29.48 | 14.68 | 28.56 | 14.59 | 24.35 | 13.61 | 20.82 |
| 3R | 15.98 | 29.55 | 17.24 | 27.90 | 13.56 | 24.82 | 12.68 | 22.74 |
| 4L | 18.32 | 28.40 | 16.68 | 29.36 | 12.03 | 22.37 | 12.06 | 20.23 |
| 4R | 17.84 | 27.33 | 17.91 | 28.78 | 14.13 | 23.22 | 13.34 | 21.03 |
| 5L | 16.82 | 24.80 | 15.68 | 24.44 | 12.34 | 19.21 | 12.56 | 17.40 |
| 5R | 17.15 | 26.08 | 15.87 | 26.10 | 12.25 | 17.90 | 11.63 | 16.85 |
| 6L | 17.97 | 27.77 | 18.88 | 27.01 | 11.72 | 22.68 | 9.97 | 18.59 |
| 6R | 16.43 | 28.77 | 16.20 | 26.64 | 12.93 | 21.47 | 11.99 | 21.77 |
| 7L | 17.44 | 29.45 | 15.36 | 28.30 | 11.39 | 20.72 | 10.98 | 18.77 |
| 7R | 19.46 | 28.25 | 17.69 | 29.02 | 11.78 | 20.54 | 12.25 | 20.21 |
| 8L | 18.24 | 29.86 | 17.79 | 28.43 | 12.67 | 21.71 | 12.91 | 22.32 |
| 8R | 18.06 | 29.54 | 17.48 | 29.16 | 11.86 | 22.75 | 12.21 | 22.32 |
| 9L | 21.07 | 29.88 | 18.80 | 29.39 | 15.34 | 25.61 | 13.57 | 23.99 |
| 9R | 21.32 | 29.64 | 19.37 | 28.06 | 14.60 | 25.10 | 13.80 | 23.91 |
| 10L | 15.89 | 32.58 | 16.51 | 29.57 | 13.37 | 22.18 | 13.64 | 19.64 |
| 10R | 14.26 | 29.73 | 15.75 | 28.81 | 13.65 | 21.15 | 12.65 | 20.73 |
| 11L | 17.28 | 30.79 | 17.57 | 30.18 | 11.71 | 19.97 | 10.99 | 17.65 |
| 11R | 16.73 | 30.54 | 18.66 | 29.02 | 12.57 | 17.74 | 11.25 | 18.36 |
| 12L | 21.51 | 30.03 | 21.09 | 30.68 | 13.33 | 20.00 | 11.14 | 19.71 |
| 12R | 22.00 | 31.26 | 19.98 | 32.38 | 14.34 | 22.21 | 12.63 | 21.64 |

Table A-3: Average Gaussian curvature data for the full articular surface on each specimen

|  | Medial Cuneiform | First Metatarsal | Intermediate Cuneiform | Second Metatarsal |
| --- | --- | --- | --- | --- |
| 1L | -0.0294 | -0.0201 | 0.0540 | 0.0771 |
| 1R | -0.0175 | -0.0160 | 0.0538 | 0.0206 |
| 2L | -0.0023 | 0.0050 | -0.0194 | 0.0141 |
| 2R | 0.0004 | -0.0033 | -0.0173 | 0.0142 |
| 3L | 0.0018 | 0.0092 | 0.0049 | 0.0280 |
| 3R | 0.0040 | 0.0045 | 0.0210 | 0.0300 |
| 4L | 0.0022 | 0.0160 | -0.0053 | 0.0081 |
| 4R | 0.0072 | 0.0221 | 0.0102 | 0.0297 |
| 5L | 0.0095 | 0.0280 | 0.0155 | 0.0220 |
| 5R | 0.0251 | 0.0248 | -0.0259 | 0.0165 |
| 6L | -0.0208 | -0.0147 | -0.0200 | -0.0009 |
| 6R | -0.0065 | 0.0037 | 0.0209 | 0.0048 |
| 7L | -0.0246 | -0.0141 | -0.0310 | -0.0184 |
| 7R | -0.0194 | -0.0059 | -0.0185 | 0.0130 |
| 8L | 0.0110 | 0.0303 | 0.0115 | 0.0199 |
| 8R | 0.0053 | 0.0213 | 0.0099 | 0.0151 |
| 9L | -0.0026 | -0.0004 | 0.0092 | 0.0189 |
| 9R | -0.0116 | -0.0169 | 0.0242 | 0.0326 |
| 10L | 0.0312 | 0.0140 | 0.0280 | 0.0425 |
| 10R | 0.0120 | 0.0263 | 0.0288 | 0.0473 |
| 11L | 0.0009 | -0.0099 | 0.0303 | 0.0336 |
| 11R | -0.0072 | -0.0066 | 0.0301 | 0.0499 |
| 12L | 0.0209 | 0.0115 | 0.0176 | 0.0303 |
| 12R | 0.0260 | 0.0193 | 0.0106 | 0.0266 |

Table A-4: Average Gaussian curvature for each subregion of each surface on each specimen

| Medial Cuneiform | | | | |
| --- | --- | --- | --- | --- |
|  | Dorsal-Medial | Plantar-Medial | Plantar-Lateral | Dorsal-Lateral |
| 1L | -0.0046 | 0.0488 | 0.0142 | 0.0071 |
| 1R | -0.0274 | 0.0047 | 0.0246 | 0.0227 |
| 2L | 0.0022 | -0.0515 | -0.0251 | 0.0063 |
| 2R | -0.0344 | 0.0265 | 0.0030 | -0.0104 |
| 3L | -0.0007 | 0.0204 | 0.0280 | 0.0273 |
| 3R | 0.0178 | 0.0178 | 0.0266 | 0.0250 |
| 4L | 0.0103 | 0.0147 | 0.0038 | 0.0296 |
| 4R | 0.0002 | -0.0016 | 0.0209 | 0.0152 |
| 5L | 0.0364 | 0.0468 | 0.0318 | 0.0381 |
| 5R | 0.0377 | -0.0160 | 0.0133 | 0.0462 |
| 6L | -0.0088 | -0.0328 | -0.0581 | 0.0314 |
| 6R | 0.0205 | -0.0400 | -0.0343 | 0.0387 |
| 7L | -0.0029 | -0.0836 | 0.0424 | -0.0066 |
| 7R | -0.0527 | -0.0007 | 0.0029 | -0.0301 |
| 8L | 0.0422 | 0.0334 | 0.0368 | 0.0257 |
| 8R | 0.0275 | -0.0031 | 0.0164 | 0.0244 |
| 9L | 0.0326 | -0.0340 | -0.0037 | 0.0431 |
| 9R | -0.0023 | -0.0314 | -0.0474 | 0.0241 |
| 10L | 0.0352 | 0.0148 | 0.0786 | 0.0334 |
| 10R | 0.0268 | 0.0297 | 0.0351 | 0.0253 |
| 11L | -0.0101 | 0.0201 | -0.0414 | 0.0140 |
| 11R | -0.0068 | -0.0401 | -0.0372 | 0.0285 |
| 12L | 0.0304 | 0.0426 | -0.0155 | 0.0201 |
| 12R | 0.0334 | 0.0269 | 0.0330 | 0.0197 |
| First Metatarsal | | | | |
|  | Dorsal-Medial | Plantar-Medial | Plantar-Lateral | Dorsal-Lateral |
| 1L | 0.0370 | -0.0059 | 0.0266 | 0.0690 |
| 1R | -0.0221 | 0.0281 | 0.0269 | 0.0451 |
| 2L | -0.0317 | 0.0290 | -0.0184 | 0.0283 |
| 2R | 0.0087 | -0.0012 | 0.0056 | 0.0146 |
| 3L | 0.0177 | -0.0259 | 0.0243 | 0.0317 |
| 3R | 0.0313 | -0.0242 | 0.0312 | 0.0305 |
| 4L | -0.0248 | -0.0178 | 0.0177 | 0.0340 |
| 4R | 0.0134 | -0.0231 | 0.0315 | 0.0019 |
| 5L | 0.0559 | 0.0214 | 0.0566 | 0.0349 |
| 5R | 0.0626 | -0.0094 | 0.0127 | 0.0710 |
| 6L | -0.0329 | -0.0308 | -0.0608 | 0.0396 |
| 6R | -0.0076 | -0.0016 | 0.0057 | 0.0607 |
| 7L | -0.0216 | -0.0064 | 0.0283 | -0.0005 |
| 7R | -0.0478 | -0.0114 | 0.0731 | 0.0310 |
| 8L | 0.0151 | 0.0048 | 0.0670 | 0.0549 |
| 8R | 0.0354 | 0.0016 | 0.0712 | 0.0582 |
| 9L | 0.0281 | -0.0257 | -0.0375 | 0.0386 |
| 9R | -0.0026 | -0.0277 | -0.0217 | 0.0266 |
| 10L | 0.0376 | -0.0618 | 0.0755 | 0.0516 |
| 10R | 0.0355 | 0.0569 | 0.0886 | 0.0418 |
| 11L | -0.0396 | 0.0313 | 0.0446 | 0.0349 |
| 11R | -0.0462 | -0.0299 | 0.0265 | 0.0356 |
| 12L | 0.0517 | 0.0005 | -0.0238 | 0.0265 |
| 12R | 0.0440 | 0.0248 | -0.0098 | 0.0399 |
| Intermediate Cuneiform | | | | |
|  | Dorsal-Medial | Plantar-Medial | Plantar-Lateral | Dorsal-Lateral |
| 1L | -0.0451 | -0.0428 | 0.0717 | -0.0521 |
| 1R | -0.0087 | -0.0004 | 0.0687 | 0.0261 |
| 2L | -0.0307 | -0.0326 | -0.0028 | -0.0440 |
| 2R | -0.0757 | -0.0505 | 0.0054 | -0.0464 |
| 3L | -0.0062 | 0.0132 | 0.0330 | -0.0054 |
| 3R | -0.0035 | 0.0521 | 0.0291 | -0.0173 |
| 4L | -0.0458 | -0.0007 | -0.0434 | -0.0285 |
| 4R | -0.0396 | -0.0189 | 0.0168 | -0.0244 |
| 5L | 0.0154 | -0.0455 | 0.0452 | -0.0262 |
| 5R | 0.0531 | -0.0610 | -0.0486 | -0.0207 |
| 6L | -0.0164 | 0.0126 | -0.0174 | 0.0213 |
| 6R | -0.0304 | 0.0358 | -0.0202 | -0.0318 |
| 7L | -0.0393 | -0.0259 | -0.0326 | 0.0327 |
| 7R | 0.0201 | -0.0241 | -0.0348 | 0.0205 |
| 8L | 0.0066 | 0.0244 | -0.0519 | -0.0201 |
| 8R | -0.0187 | 0.0410 | -0.0646 | 0.0171 |
| 9L | -0.0115 | -0.0241 | -0.0181 | -0.0230 |
| 9R | 0.0126 | 0.0085 | 0.0213 | 0.0192 |
| 10L | 0.0163 | 0.0199 | -0.0009 | 0.0580 |
| 10R | 0.0170 | 0.0111 | -0.0407 | 0.0331 |
| 11L | -0.0253 | 0.0093 | 0.0351 | -0.0274 |
| 11R | -0.0303 | 0.0358 | 0.0579 | -0.0516 |
| 12L | 0.0203 | -0.0329 | 0.0753 | -0.0364 |
| 12R | -0.0152 | -0.0188 | -0.0476 | -0.0417 |
| Second Metatarsal | | | | |
|  | Dorsal-Medial | Plantar-Medial | Plantar-Lateral | Dorsal-Lateral |
| 1L | 0.0369 | -0.0251 | 0.0767 | 0.0328 |
| 1R | 0.0025 | -0.0519 | -0.1669 | 0.0253 |
| 2L | -0.0256 | -0.0253 | -0.0287 | -0.4540 |
| 2R | -0.0311 | -0.0178 | -0.0334 | -0.0514 |
| 3L | -0.0449 | -0.0074 | 0.0595 | 0.0198 |
| 3R | -0.0271 | 0.0269 | 0.0303 | -0.0163 |
| 4L | -0.0630 | 0.0137 | -0.0223 | -0.0024 |
| 4R | -0.0485 | 0.0295 | 0.0399 | 0.0235 |
| 5L | -0.0502 | -0.0657 | 0.0286 | 0.0390 |
| 5R | -0.0532 | -0.0727 | -0.0630 | -0.0805 |
| 6L | -0.0596 | -0.0365 | -0.0902 | 0.0754 |
| 6R | -0.0295 | 0.0448 | 0.0601 | -0.0910 |
| 7L | -0.0227 | -0.0445 | -0.0494 | -0.0241 |
| 7R | -0.0358 | -0.0504 | 0.0444 | 0.0207 |
| 8L | -0.0222 | -0.0114 | -0.0381 | -0.0243 |
| 8R | -0.0249 | 0.0320 | -0.0866 | 0.0358 |
| 9L | -0.0201 | -0.0067 | -0.0113 | -0.0209 |
| 9R | -0.0229 | 0.0203 | 0.0089 | -0.0023 |
| 10L | 0.0106 | -0.0096 | -0.0309 | 0.0614 |
| 10R | -0.0219 | -0.0131 | -0.0259 | 0.0599 |
| 11L | -0.0230 | 0.0104 | 0.0425 | -0.0644 |
| 11R | -0.0174 | -0.0143 | 0.0660 | -0.0130 |
| 12L | -0.0725 | -0.0339 | 0.0364 | 0.0562 |
| 12R | -0.0635 | -0.0009 | -0.0394 | 0.0357 |

Table A-5: Calculated curvature similarity values for the full articular surface of each TMT joint on all specimens.

|  | First TMT Joint | Second TMT Joint |
| --- | --- | --- |
| 1L | -0.8583 | -0.8661 |
| 1R | -0.9625 | -0.7058 |
| 2L | 0.7478 | 0.8783 |
| 2R | 0.5218 | 0.9210 |
| 3L | -0.5853 | -0.5692 |
| 3R | -0.9513 | -0.8659 |
| 4L | -0.5371 | 0.8444 |
| 4R | -0.6725 | -0.6830 |
| 5L | -0.6805 | -0.8680 |
| 5R | -0.9948 | 0.8362 |
| 6L | -0.8690 | -0.4261 |
| 6R | 0.8034 | -0.6102 |
| 7L | -0.8053 | -0.8153 |
| 7R | -0.6592 | 0.8671 |
| 8L | -0.6944 | -0.8077 |
| 8R | -0.6234 | -0.8451 |
| 9L | -0.5516 | -0.7618 |
| 9R | -0.8595 | -0.8854 |
| 10L | -0.7418 | -0.8466 |
| 10R | -0.7458 | -0.8227 |
| 11L | 0.4899 | -0.9570 |
| 11R | -0.9636 | -0.8200 |
| 12L | -0.7940 | -0.8091 |
| 12R | -0.8854 | -0.7145 |

Table A-6: Calculated curvature similarity values for each subregion of both TMT joints in each specimen

| First TMT Joint | | | | |
| --- | --- | --- | --- | --- |
|  | Dorsal-Medial | Plantar-Medial | Plantar-Lateral | Dorsal-Lateral |
| 1L | 0.5248 | 0.5215 | -0.7858 | -0.5031 |
| 1R | -0.9146 | -0.5629 | -0.9626 | -0.7703 |
| 2L | 0.4633 | 0.8004 | -0.8812 | -0.6052 |
| 2R | 0.6262 | 0.4266 | -0.7867 | 0.8716 |
| 3L | 0.4162 | 0.9061 | -0.9420 | -0.9391 |
| 3R | -0.8031 | 0.8823 | -0.9352 | -0.9205 |
| 4L | 0.7238 | 0.9233 | -0.5995 | -0.9432 |
| 4R | -0.3538 | -0.4631 | -0.8488 | -0.5255 |
| 5L | -0.8429 | -0.7464 | -0.7998 | -0.9633 |
| 5R | -0.8195 | -0.8124 | -0.9803 | -0.8427 |
| 6L | -0.6358 | -0.9734 | -0.9807 | -0.9085 |
| 6R | 0.6988 | -0.4170 | 0.5620 | -0.8365 |
| 7L | -0.5342 | -0.4726 | -0.8506 | -0.4716 |
| 7R | -0.9593 | -0.4521 | -0.4164 | 0.9874 |
| 8L | -0.6914 | -0.5427 | -0.7935 | -0.7521 |
| 8R | -0.9012 | 0.7769 | -0.6106 | -0.7259 |
| 9L | -0.9394 | -0.8916 | -0.4983 | -0.9543 |
| 9R | -0.9494 | -0.9484 | -0.7466 | -0.9589 |
| 10L | -0.9722 | 0.6170 | -0.9828 | -0.8411 |
| 10R | -0.8912 | -0.7798 | -0.7132 | -0.8210 |
| 11L | -0.6276 | -0.8387 | 0.9687 | -0.7160 |
| 11R | -0.5458 | -0.8869 | 0.8716 | -0.9119 |
| 12L | -0.8126 | -0.3412 | -0.8430 | -0.8928 |
| 12R | -0.8931 | -0.9659 | 0.6548 | -0.7654 |
| Second TMT Joint | | | | |
|  | Dorsal-Medial | Plantar-Medial | Plantar-Lateral | Dorsal-Lateral |
| 1L | 0.9198 | -0.8118 | -0.9716 | 0.8327 |
| 1R | 0.6487 | -0.3212 | 0.7218 | -0.9867 |
| 2L | -0.9269 | -0.9008 | -0.4973 | -0.4966 |
| 2R | -0.7213 | -0.6883 | 0.5582 | -0.9574 |
| 3L | -0.5377 | 0.7991 | -0.7962 | 0.6393 |
| 3R | -0.5294 | -0.7769 | -0.9828 | -0.9748 |
| 4L | -0.8784 | 0.4364 | -0.7757 | -0.4820 |
| 4R | -0.9191 | 0.8380 | -0.7269 | 0.9839 |
| 5L | 0.6609 | -0.8624 | -0.8342 | 0.8527 |
| 5R | 0.9992 | -0.9292 | -0.8987 | -0.6290 |
| 6L | -0.6409 | 0.6840 | -0.5832 | -0.6456 |
| 6R | -0.9871 | -0.9112 | 0.6786 | -0.6865 |
| 7L | -0.8075 | -0.8097 | -0.8471 | 0.8830 |
| 7R | 0.7996 | -0.7573 | 0.9043 | -0.9958 |
| 8L | 0.6550 | 0.7516 | -0.8816 | -0.9239 |
| 8R | -0.8894 | -0.9028 | -0.8871 | -0.7571 |
| 9L | -0.8048 | -0.6427 | -0.8302 | -0.9601 |
| 9R | 0.7940 | -0.7256 | -0.7252 | 0.5204 |
| 10L | -0.8425 | 0.7595 | -0.3944 | -0.9759 |
| 10R | 0.9009 | 0.9329 | -0.8359 | -0.7952 |
| 11L | -0.9603 | -0.9537 | -0.9233 | -0.7293 |
| 11R | -0.8059 | 0.7150 | -0.9462 | -0.6255 |
| 12L | 0.6440 | -0.9872 | -0.7601 | 0.8413 |
| 12R | -0.6169 | -0.4311 | -0.9241 | 0.9368 |

Supplement B: P-values from statistical analyses

Table B-1: P-values from paired t-tests comparing articular surface area, maximum width, and maximum height between opposing joint surfaces in the first and second TMT joints. Asterisk and yellow highlight show statistical significance.

|  | Surface Area | Width | Height |
| --- | --- | --- | --- |
| First TMT Joint | 0.786 | 0.022* | 0.007* |
| Second TMT Joint | 0.002* | <0.001* | <0.001* |

Table B-2: P-values from one-subject t-tests comparing curvature values to zero for each whole surface and subregion of each articular surface. Asterisk and yellow highlight show statistical significance.

|  | Whole Surface | Dorsal-Medial | Plantar-Medial | Plantar-Lateral | Dorsal-Lateral |
| --- | --- | --- | --- | --- | --- |
| Medial Cuneiform | 0.845 | 0.113 | 0.943 | 0.374 | <0.001* |
| First Metatarsal | 0.044* | 0.257 | 0.427 | 0.009* | <0.001* |
| Intermediate Cuneiform | 0.117 | 0.057 | 0.466 | 0.867 | 0.095 |
| Second Metatarsal | <0.001* | <0.001* | 0.057 | 0.515 | 0.487 |

Table B-3: P-values from one-subject t-tests comparing curvature similarity values to positive and negative one and one-sided one-subject t-tests evaluating if each curvature similarity is less than zero for each whole joint and subregion of each joint. Asterisk and yellow highlight show statistical significance.

|  | | Whole Joint | Dorsal-Medial | Plantar-Medial | Plantar-Lateral | Dorsal-Lateral |
| --- | --- | --- | --- | --- | --- | --- |
| First TMT | H_0_ = 1 | <0.001* | <0.001* | <0.001* | <0.001* | <0.001* |
|  | H_0_ = -1 | <0.001* | <0.001* | <0.001* | 0.001* | 0.003* |
|  | Less than 0 | <0.001* | 0.001* | 0.075 | <0.001* | <0.001* |
| Second TMT | H_0_ = 1 | <0.001* | <0.001* | <0.001* | <0.001* | <0.001* |
|  | H_0_ = -1 | 0.001* | <0.001* | <0.001* | 0.001* | <0.001* |
|  | Less than 0 | 0.003* | 0.112 | 0.045* | <0.001* | 0.063 |

Table B-4: P-values from Games-Howell post-hoc analysis following three-way ANOVA for differences in curvature between whole articular surfaces. Asterisk and yellow highlight show statistical significance.

| **Comparison Bone 1** | **Comparison Bone 2** | **P-value** |
| --- | --- | --- |
| Medial Cuneiform | Intermediate Cuneiform | 0.368 |
| Medial Cuneiform | First Metatarsal | 0.745 |
| Medial Cuneiform | Second Metatarsal | <0.001* |
| Intermediate Cuneiform | First Metatarsal | 0.839 |
| Intermediate Cuneiform | Second Metatarsal | 0.122 |
| First Metatarsal | Second Metatarsal | 0.003* |

Table B-5: P-values from Games-Howell post-hoc analysis following three-way ANOVA for differences in curvature between articular surface subregions within each bone. Asterisk and yellow highlight show statistical significance.

Region definitions: 1 – Dorsal-Medial, 2 – Plantar-Medial, 3 – Plantar-Lateral, 4 – Dorsal-Lateral

| **Comparison** | **Medial Cuneiform** | **First Metatarsal** | **Intermediate Cuneiform** | **Second Metatarsal** |
| --- | --- | --- | --- | --- |
| 1:2 | 0.802 | 0.498 | 0.855 | 0.159 |
| 1:3 | 0.994 | 0.531 | 0.602 | 0.341 |
| 1:4 | 0.214 | 0.004* | 1.000 | 0.893 |
| 2:3 | 0.938 | 0.035* | 0.940 | 0.985 |
| 2:4 | 0.070 | <0.001* | 0.894 | 1.000 |
| 3:4 | 0.250 | 0.327 | 0.654 | 0.992 |

Table B-6: P-values from Games-Howell post-hoc analysis following three-way ANOVA for differences in curvature similarity between subregions within each TMT joint. Asterisk and yellow highlight show statistical significance.

Region definitions: 1 – Dorsal-Medial, 2 – Plantar-Medial, 3 – Plantar-Lateral, 4 – Dorsal-Lateral

| **Comparison** | **First TMT Joint** | **Second TMT Joint** |
| --- | --- | --- |
| 1:2 | 0.655 | 0.990 |
| 1:3 | 0.952 | 0.329 |
| 1:4 | 0.576 | 0.995 |
| 2:3 | 0.360 | 0.035* |
| 2:4 | 0.087 | 1.000 |
| 3:4 | 0.890 | 0.476 |
